# Prevalence of Sexual Abuse in Adults with Intellectual Disability: Systematic Review and Meta-Analysis

**DOI:** 10.3390/ijerph18041980

**Published:** 2021-02-18

**Authors:** Raluca Tomsa, Smaranda Gutu, Daniel Cojocaru, Belén Gutiérrez-Bermejo, Noelia Flores, Cristina Jenaro

**Affiliations:** 1 Department of Psychology, University of Bucharest, 050663 Bucharest, Romania; smaranda.gutu@drd.unibuc.ro (S.G.); daniel.cojocaru@drd.unibuc.ro (D.C.); 2 PROTEDIS/Faculty of Psychology, Universidad Nacional de Educación a Distancia (UNED), 28040 Madrid, Spain; mbgutierrez@psi.uned.es; 3 INICO/Faculty of Psychology, Universidad de Salamanca, 37005 Salamanca, Spain; nrobaina@usal.es

**Keywords:** intellectual disability, sexual abuse, adults, prevalence, systematic review, meta-analysis

## Abstract

This study presents the results of a systematic review on the prevalence of sexual abuse experienced in adulthood by individuals with intellectual disability. An electronic and manual search of academic journals was performed on four databases via EBSCO Host: Academic Search Complete, PsycINFO, Medline, CINAHL Full-Text. In addition, PubMed, ProQuest, and Web of Science (core collection) were searched. After an initial selection of 1037 documents, 25 articles remained for quantitative synthesis. The combined prevalence of sexual abuse in adults with intellectual disability was 32.9% (95% CI: 22.7–43.0) and sensitivity analysis revealed that the prevalence was not outweighed by a single study. Overall, the United Kingdom had the highest prevalence (r = 34.1%), and the USA had the lowest (r = 15.2%). The overall prevalence in females was lower (r = 31.8%) than that in males (r = 39.9%). Subgroup analyses revealed that prevalence of sexual abuse was higher in institutionalized individuals. The most prevalent profile of abuser is of a peer with intellectual disability. Prevalence increases from mild to severe levels of intellectual disability and decreases in profound levels. It is also more prevalent when the informant is the individual with intellectual disability than when someone else reports abuse. In sum, one in three adults with intellectual disability suffers sexual abuse in adulthood. Special attention should be paid for early detection and intervention in high risk situations.

## 1. Introduction

The Convention on the Rights of Persons with Disabilities from the United Nations (UN-CRDP) states in article 16.1 that “States Parties shall take all appropriate (…) measures to protect persons with disabilities (…) from all forms of exploitation, violence and abuse, including their gender-based aspects” [1]. Despite the existence of these and other regulations that protect the rights of this group at a national and international level, there is still a long way to go to achieve equal opportunities [2].

Intellectual disability includes different conditions classifiable according to the International Classification of Diseases ICD-11 [3] as disorders of intellectual development (6A00). These disorders are a group of etiologically diverse conditions originating during the developmental period characterized by significantly below average intellectual functioning and adaptive behavior. A similar definition is included in the Diagnostic and Statistical Manual of Mental Disorders, DSM-5 manual [4] where the condition is called intellectual disability. According to the International Classification of Functioning, Disability and Health (ICF), this condition is related to limitations in general mental functions, required to understand and constructively integrate the various mental functions including all cognitive functions and their development over the life span [5]. Finally, The American Association on Intellectual and Developmental Disabilities [6] defines it as a disability characterized by significant limitations in both intellectual functioning and in adaptive behavior that covers many everyday social and practical skills. This disability originates before the age of 18. All these definitions share common elements in considering that this condition implies limitations that require support to guarantee the same rights as for the general population. As we pointed out before, one of these rights is related to the guarantees of protection and avoidance of abuse in sexual matters.

Sexual abuse can be defined as unwanted sexual activity, with perpetrators using force, bribes or coercion, making threats or taking advantage of victims who are unable to give consent by virtue of age, immaturity or intellect [7]. It is also considered sexual abuse when one person exposes his/her genitals or looks at or touches certain parts of another’s body for the purpose of gratifying or satisfying the needs of the first person. Sexual offense may also include exposing one’s genital area to another person and/or compelling that person to look at or touch the above mentioned parts of the first person’s body when a barrier to consent is present for that person [8].

In contrast to individuals with average IQ or above, individuals with intellectual disability (ID) are more likely to experience sexual abuse [9], and less likely to report it [10,11]. A literature review suggests that from 7% to 34% individuals with ID have experienced sexual abuse in adult life [7]. This range is even wider in other studies and this is problematic. There are several issues related to the study of this topic, such as the different definitions of sexual abuse and the difficulty in accessing the information due to the characteristics of the informants who, in many cases, do not know or cannot or do not wish to report these situations. As an example, these adults have more difficulties in reporting their experiences of sexual assault [12]. Despite methodological and definitional difficulties, there is no doubt that people with intellectual disability are more vulnerable to sexual abuse than the general population [13].

Qualitative synthesis has shown that sexual abuse in individuals with ID has a broad range of psychological, behavioral, and social consequences. Conduct disorders, self-injury, inappropriate sexual talk and poor feelings of personal safety seem to be more indicative for the ID population [7]. Anxiety, depression and PTSD (post-traumatic stress disorder) are prevalent in individuals who have experienced sexual abuse [14]. While there is more scientific evidence on sexual abuse in children and in those with intellectual disability, as well as on consequences in adulthood of sexual abuse in childhood [7,14], the information specifically focused on sexual abuse in adults with intellectual disability, and associated contextual and personal variables is scarce. Existing systematic reviews focus on sexual abuse experienced during childhood and adulthood [7,14]. Other systematic reviews analyze sexuality issues from a broader perspective, including information, skills, interests, and negative experiences [15,16].

Previous research has shown that some of the variables associated with higher risk of experiencing sexual abuse are living in residential settings or being institutionalized [17,18], being female [19,20,21,22], being a child or adolescent with intellectual disability [14], and having high severity of support needs [23]. As mentioned, earlier narrative reviews also highlight the difficulties in estimating the rates of sexual abuse in this population, and the substantial differences among these rates [14]. For example, in the narrative review from Byrne [14], prevalence rates ranged from 14% to 32% for children with intellectual disability and from 7% to 34% for adults with intellectual disability. A recent systematic review of qualitative studies on adults with intellectual disability who have been victims of sexual assault demonstrates that they face additional internal and external barriers when reporting these incidents [12].

Concerning individual risk factors for sexual abuse, previous reviews with diverse samples in terms of age, disability, and severity, suggest that women [14,19], young children, and severity of intellectual disability between mild-moderate ranges constitute risk factors [14]. Regarding the profile of the abusers, studies suggest that the offender is generally male [19]. Concerning environmental factors, a systematic review on social vulnerability of this population [9] revealed that living in congregate settings, as well as greater community participation, together with inappropriate training on sexual matters, increases the chances of victimization [9].

Although all these studies provide interesting evidence, there are difficulties when it comes to comparing studies, assessing their quality, and identifying factors associated with the variability of the findings. Systematic reviews address the need to access high quality, relevant, accessible, and up-to-date information on a topic of interest [24]. To further study sexual abuse experienced in adult life in individuals with intellectual disability and associated variables, systematic approaches and quantitative synthesis (meta-analysis) are required.

Specifically, the purpose of this study is to conduct a systematic review and meta-analysis of studies on prevalence of sexual abuse in adults with intellectual disability. Thus, the aims are: (1) to examine differences in prevalence associated with individual characteristics (gender, severity of the intellectual disability); (2) to examine differences in prevalence associated with contextual characteristics (country, period of publication of the study, place where the sexual abuse happened, profile of the abuser; informant of the abuse); (3) to analyze the moderator effect of age on prevalence.

## 2. Materials and Methods

The review followed the Preferred Reporting Items for Systematic Reviews and Meta-Analyses (PRISMA) Statement [25]. The protocol of the current systematic review is available through International Prospective Register of Systematic Reviews (PROSPERO) (https://www.crd.york.ac.uk/prospero/, accessed on 29 January 2021, registered date: 29 January 2021); registration number: CRD42021228292.

### 2.1. Search and Selection Strategy

An electronic search was conducted in seven databases: PsycINFO, Medline, PubMed, CINAHL Full-Text, ProQuest, Web of Science, and Academic Search Complete. The search strategy for all databases included terms included in the Medical Subject Headings. In order to find studies not detected by the electronic search, a manual search was performed reviewing reference lists of eligible studies, as well as searching in the “most-likely to publish” editorial webs (Emerald, MDPI, Science Direct, Springer, SAGE Publishing, Taylor & Francis, and Wiley). Searches were made using the following keywords or their combination accessed on 1 November 2020: (‘(“intellectual disability” (MeSH Terms) OR “intellectual disability” (MeSH Terms) OR “learning disabilities” (MeSH Terms) OR “developmental disabilities” (MeSH Terms) OR “learning disabilities” (MeSH Terms)) AND (“rape” (MeSH Terms) OR “sex offenses” (MeSH Terms) OR “sex offenses” (MeSH Terms))).

**Figure 1:**
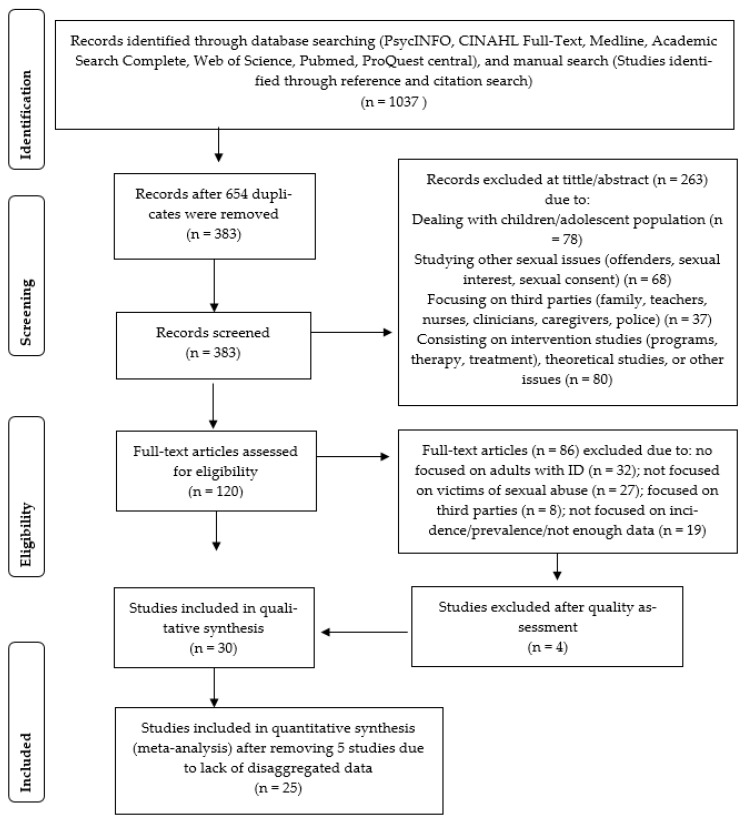
PRISMA flowchart of systematic review process.

Four authors (R.T., S.G, D.C., and C.J,) conducted a two-step literature search, in order to assess the articles for eligibility criteria. During the first stage, studies were examined with regard to inclusion criteria after reading the title and the abstract. During this stage, studies were retained when there was no agreement on inclusion between the reviewers. During the second stage, the remaining studies were assessed on eligibility criteria after reading the full-text. After data collection and extraction (during which the authors were blind to each other’s results), the appointed authors compared their results to reach a final consensus based on consensual inclusion and exclusion criteria. Potential discrepancy in the judgment was addressed during meetings with two additional authors (B.G.B. and N.F.) with the aim to obtain a shared pool of included studies for the meta-analysis. In all, a total of 30 articles were retained for qualitative analysis (Figure 1).

### 2.2. Inclusion and Exclusion Criteria

A modified version of the PICOS (i.e., Participants-Intervention-Comparison-Outcomes) approach for observational cross-sectional studies, as defined in the PRISMA guidelines [26], was used. Criteria for inclusion of studies were the following.

(a) Characteristics of participants. Studies were included if they were conducted on or included adults (18+) with Intellectual and/or Developmental Disabilities (ID/DD) as defined by the ICD [3], DSM [4], or the AAIDD [6]. Participants must have experienced sexual abuse; this ranges from verbal harassment or unwanted sexual advances to forced penetration, and an array of types of coercion, from social pressure and intimidation to physical force. Behaviors such as exposing the genitals or looking at or touching certain parts of a victim’s body or requiring the victim to perform sexual acts are defined as sexual abuse. Several characteristics must apply to the victim: (1) he/she withholds the consent, or (2) is unable to give informed consent to, or is not developmentally prepared, or (3) the victim is unduly pressured due to the relationship (e.g., familial), the use of force, a weapon, or threats. Studies were included if they referred to adult samples, and if samples were over 10 subjects.
(b) Characteristics of intervention, factor, or exposure. Studies were included if they reported data of sexual abuse.
(c) Characteristics of comparison or control. For the meta-analysis, studies were included if they used control groups with intellectual disability who were not victims of sexual abuse. They also were included if they used comparison groups from the general population consisting of participants without intellectual disability who were victims of sexual abuse.
(d) Characteristics of outcome. Studies reported prevalence of sexual abuse on population (total, females, and males) with intellectual disability.

### 2.3. Quality Assessment

The appraisal of individual study quality, depending on the type of study, was based on tailored quality assessment tools from the National Health, Lung, and Blood Institute (NHLBI) of the National Institutes of Health (NIH): (1) The Quality Assessment Tool for Observational Cohort and Cross-Sectional Studies and (2) the Quality Assessment Tool for Case-Control Studies. The tools were designed to assist reviewers in focusing on concepts that are central for critical appraisal of the internal validity of a study. Each of the quality assessment tools has a detailed guidance document that provides more detailed descriptions and examples of application of the items, as well as justifications for each item’s inclusion. For some items, examples are provided to clarify the intent of the question and the appropriate rater response. The rating tools allow judging for each study to be of “good,” “fair,” or “poor” quality. The ratings on the different items are used to assess the risk of bias in the study due to flaws in study design or implementation. In general terms, a “good” study has the least risk of bias, and results are considered to be valid. A “fair” study is susceptible to some bias, but deemed not sufficient to invalidate its results. The fair quality category is likely to be broad, so studies with this rating will vary in their strengths and weaknesses. A “poor” rating indicates significant risk of bias. Studies rated poor were excluded from further analyses (See Appendix A, Table A1 for additional information).

Unlike well-known tools such as PRISMA [25] and STROBE [27] that provide a standardized guide for carrying out systematic reviews, including constructing a protocol, testing for bias and heterogeneity, and other aspects of the review process, the Quality Assessment Tools are used to assess and rate the quality of individual articles that are included in a systematic review or meta-analysis. Evaluated data were handled and compared by four of the authors (R.T., S.G., D.C., and C.J.). Disagreement between the independent raters was resolved by consensus among them.

## 3. Results

### 3.1. Qualitative Synthesis

The 30 studies included in the review that explicitly addressed the study objectives are contained in Table 1. The United Kingdom (*n* = 9) conducted the most studies. Next was the United States of America (USA) (*n* = 7), followed by the Republic of Ireland (*n* = 2), South Africa (*n* = 2), Spain (*n* = 2), Sweden (*n* = 2), and Taiwan (*n* = 2). Each of the following countries had one study: Canada, Norway, Sri Lanka, and the Netherlands. The studies included sample sizes of sexually abused adults with intellectual disability ranging from 8 to 1071 (M = 114.5). The whole sample was comprised of 3434 abused adults with intellectual disability. Comparison samples ranged from 13 to 11,878 participants (M = 1076.6; N = 32,298). A total of 23 studies (76.6%) were performed in a clinical context, meaning that all participants were identified as victims with intellectual disability or alleged victims of sexual abuse, or the participants came from reported incidents of sexual abuse, or were attending an intervention program. These are convenience samples rather than samples drawn from the general population. In some cases, the absence of comparison data prevents the obtaining of global or sex-disaggregated prevalence data, which explains why some studies were removed from the meta-analysis. It can be noted that the studies offer prevalence data from information gathered over several years (M = 4.5; SD = 3.3). All the studies were retrospective.

Although four studies claim that they are longitudinal in nature, the data reported has a cross-sectional nature. The study from Beadle-Brown at al. [30] reported findings from one of the largest databases in the UK, collected between 1998 and 2005. However, data on people with intellectual disability were available for only eight months of 2005, so the authors utilized estimations and reported a rate of 17.3% of referrals for sexual abuse for this group. Likewise, Brown et al. [32] were carrying out an ongoing project, but the study focuses on the data from reported incidents of sexual abuse during a year. Similarly, Cambridge at al. [34] started from a larger study that examined 6148 adult protection referrals between 1998 and 2005, of which 397 were referrals for alleged sexual abuse. Global estimations and percentages of sexual abuse in men and women were utilized for prevalence. Finally, Lin et al. [45] analyze nationwide data from 2002–2007 on sexual assault and report prevalence and trends, although data on intellectual disability are not disaggregated by gender.

### 3.2. Quantitative Synthesis (Meta-Analysis)

Quantitative synthesis used 25 of the 30 studies (see Appendix A, Table A2). Separate meta-analyses were conducted for the total sample, male, and female prevalence. We used OpenMeta-Analyst [58] and Jamovi software [59] and a Bayesian random-effects model with a 95% credible interval (95% CI). We conducted sensitivity analyses, replicating the results after excluding one study, to examine the robustness of the analysis and the influence of the excluded study. Heterogeneity was assessed with I^2^ [60] and the Q [61] statistics. A value approximating to zero suggests homogeneity, and values of 25–50%, 50–75% and 75–100% represent low, medium and large heterogeneity, respectively. As expected, analyses indicated large and significant heterogeneity between the effect sizes, so the role of age as moderator was investigated. In addition, several sub-group meta-analyses were performed. Publication bias was explored with a visual inspection of the symmetry of the funnel plot.

### 3.3. Prevalence of Sexual Abuse in Adults with Intellectual Disability

**Figure 2:**
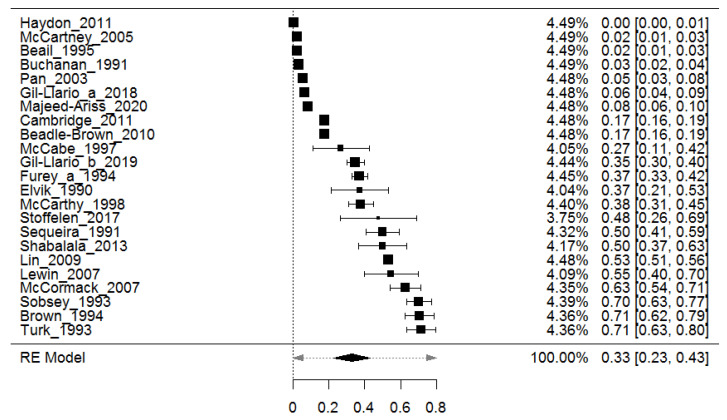
Forest plot of the prevalence of sexual abuse in adults with intellectual disability (studies sorted by observed effect sizes).

A total of 23 studies were included for the overall meta-analyses and the pooled prevalence of sexual abuse in adults with intellectual disability was 32.9% (95% CI: 22.7–43.0) (see Figure 2). The *Q* analysis showed significant results (Chi square = 5024.72, *p* < 0.001), pointing to a high heterogeneity in the included studies (I^2^ = 99.8%). We further investigated the source of heterogeneity by doing a leave-one-out sensitivity analysis to identify whether individual studies outweighed the average prevalence of sexual abuse. Our result revealed that the average obtained when each study was omitted one at a time from the analysis ranged between 32.2% (95% CI: 22.2–42.2) and 35.5% (95% CI: 25.4–45.6). This implied that the average prevalence of sexual abused among participants with intellectual disability in the different studies was not outweighed by a single study.

Since significant heterogeneity was found, age was assessed as moderator and this was not found (β = 0.001, SE = 0.01, 95% CI: −0.013–0.012, *p* = 0.982) significantly correlated to effect sizes. Results were compared according to several factors. First, more general features such as (1) country and (2) period when the study was published were analyzed.

**Figure 3:**
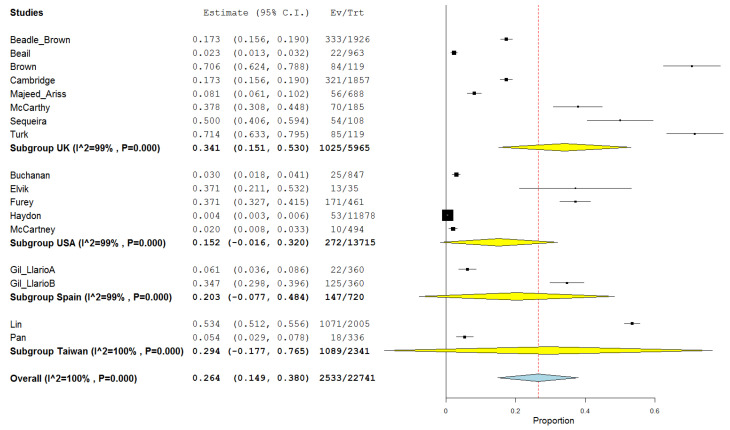
Forest plot of the comparison of the prevalence of sexual abuse in adults with intellectual disability by countries.

The overall prevalence, where more than one study was carried out in the same country, for the UK studies (n = 8) was 34.1% (95% CI: 15.1–53.0, *p* < 0.001). Prevalence in the USA studies (*n* = 5) was 15.2% (−1.6–32.0, *p* = 0.077). Prevalence in the Spain studies (*n* = 2) was 20.3% (95% CI: −7.7–48.8; *p* = 0.155) and prevalence in the Taiwan studies (*n* = 2) was 29.4% (95% CI: −17.7–76.5; *p* = 0.221) (see Figure 3).

Concerning the period when the studies were published, three groups (quartiles 25, 50, and 75, respectively) were identified: (1) up to 1994 (*n* = 9); (2) from 1995 to 2007 (*n* = 7); (3) from 2008 or later (*n* = 7). The prevalence for older studies was 48.4% (95% CI: 29.6–67.3; *p* = 0.005); for intermediate studies 26.9% (95% CI: 8.0–45.7; *p* < 0.001); and for more recent studies 25.3% (95% CI: 11.9–38.6; *p* < 0.001).

Next, more specific features of sexual abuse were analyzed: (1) setting or place(s) where the abuse happened; (2) profile of the abuser, (3) severity of the intellectual disability; (4) informant of the abuse (self-report, third parties, or both). Table 2 summarizes the results.

When comparing the results by most frequent settings where the abuse took place, four subgroups were made: (1) several places, (2) home (family home, own home, or group home with supervision or supported home, which typically consists of living in a home with two other people, or where any other small number of unrelated members live together), (3) institution (large facilities, institutionalized), and (4) service (educational, training or social services). As Table 2 summarizes, the prevalence of sexual abuse was significantly different among the four subgroups (Q = 4767.23, *p* < 0.001). Prevalence in studies where abuse took place in several places (n = 19) was 39.1% (95% CI: 21.1–57.1; *p* < 0.001); (2) prevalence in institutionalized individuals (n = 8) was 29.1% (95% CI = 15.1–43.2). In subgroup comparisons, the several places subgroup showed the highest prevalence (39.1, 95% CI: 21.1–57.1) of sexual abuse, followed, in descending order, by sexual abuse experienced in services (34.3, 95% CI: 2.3–66.3), institutions (28.1, 95% CI: 12.0–44.1), and at home (13.1%, 95% CI: −10.6–36.9).

For analyzing results by the most frequent profile of the abuser, five subgroups were made: (1) several, (2) peers (another user, roommate, partners, and friends), (3) professionals, (4) relatives, and (5) non-specified. The prevalence of sexual abuse was significantly different among the five subgroups (Q = 4767.23, *p* < 0.001) (see Table 2). The most prevalent abusers were peers (42.7%, 95% CI: 19.7–65.7), followed by relatives (36.2%, 95% CI: −30.5–100.0), several abusers (25.4%, 95% CI: 10.0–40.8), and professionals (17.6%, 95% CI: −1.9–37). Non-specified abusers obtained a prevalence of 39.2% (95% CI: 13.2–65.2).

According to the severity of the intellectual disability, four groups were made: (1) mild, (2) moderate, (3) severe, and (4) profound. As Table 2 shows, the prevalence of sexual abuse was significantly different among the four subgroups (Q = 2545.31, *p* < 0.001). The highest prevalence of sexual abuse corresponded to severe levels (67%, 95% CI: 59.5–74.4) of intellectual disability; followed by moderate (34.3%, 95%, CI: 17.2–51.4), mild (24.3%, 95% CI: 10.2–38.4); and profound (18.6%, 95%: CI: −15.7–53.0) levels. Rates of sexual abuse were significantly different among the subgroups of informants (Table 2) (Q = 4767.23, *p* < 0.001). Prevalence for self-reports was (38.0%, 95% CI: 20.1–55.8), and from someone else was 26.8% (95% CI: 9.0–44.6%). When the informant was both, prevalence was 37.4% (95% CI: 17.9–56.9).

Separate meta-analyses were conducted with male and female population (see Supplemental Material, Tables S1–S15, and Figures S1–S8). A total of 22 studies were considered for females with intellectual disability, and the prevalence of sexual abuse was 31.3% (95% CI: 8.7–43.8; Q = 2200.98, *p* < 0.001; I^2^ = 100%, T = 0.30). In clinical studies, prevalence was higher (58.4%, 95% CI: −3.9–>100) than in general or non-clinical studies (28.4%; 95% CI: 16.1–40.8). Concerning the place where the sexual abuse happened, studies with occurrence at home had the highest prevalence (45.2%, 95% CI: −42.9–>100), followed by several places (38.5%, 95% CI: 19.1–57.8), institutions (27.5%, 95% CI = 8.1–46.8), and lastly, in educational, training or social services (14%, 95% CI: −4.8–3.2). Regarding the profile of the abuser, in descending order, this consisted in peers (44.1%, 95% CI: 21.8–66.5), relatives (28.7%, 95% CI: −2.72–84.7), by several abusers (11.1%, 95% CI: 2.0–20.2), and by professionals (6.4%, 95% CI: −6.1–19.0). It should be noted that non-specified abusers had a prevalence of 62.1% (95% CI: −39.6–84.6).

A total of 16 studies were included for males with intellectual disability (see Supplemental Material), where the prevalence of sexual abuse was 39.9% (95% CI: 21.5–58.3; Q = 5620.28, *p* < 0.001; I^2^ = 99.84%, T = 0.37). In non-clinical studies, prevalence was 37.7% (95% CI: 19.0–56.4). In clinical studies, prevalence was 54.4% (95% CI: −33.0–>100.0). Grouping the studies according to place, studies where abuse occurred in institutions have the highest prevalence (50.8%, 95% CI: 15.8–85.8), followed by different places (41.8%, 95% CI: 14.1–69.4), educational, training or social services (15.9%, 95% CI: −10.2–42.1), and at home (9.8%, 95% CI: 3.3–16.2). Regarding the profile of the abuser, prevalence of sexual abuse by peers was the highest (56.1%, 95% CI: 26.3–85.9), followed by the abuse caused by several abusers (34.9%, 95% CI: 6.3–63.5), non-specified abusers (30.7%, 95% CI: −22.4–83.9) and by professionals (2.0%, 95% CI: 0.4–3.6). No studies on abuse by family members were available.

### 3.4. Publication Bias

**Figure 4:**
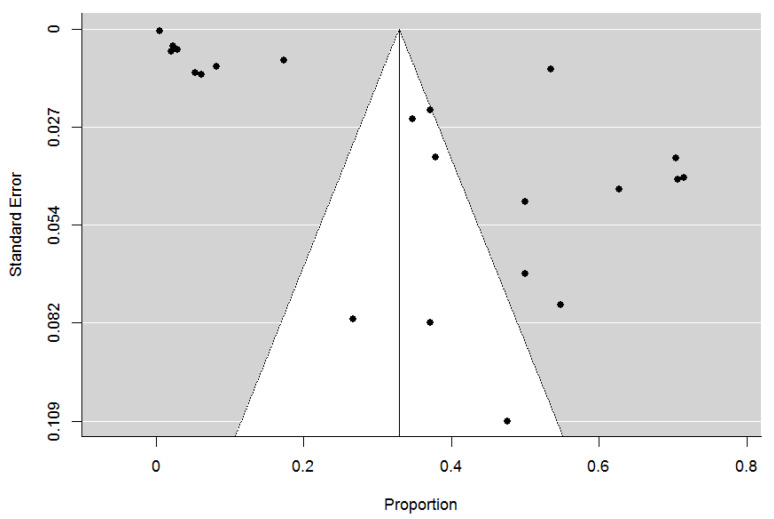
Funnel plot of publication bias on prevalence of sexual abuse in adults with intellectual disability.

Publication bias was explored with visual inspection of the funnel plot (Figure 4), which did not present significant asymmetry.

## 4. Discussion

To our knowledge, this is the first study to carry out a systematic review and meta-analysis focused on the prevalence of sexual abuse that occurs in an adult population with intellectual disability. Most of the existing reviews on the subject are narrative rather than systematic, and meta-analysis studies are lacking. To help complete the available evidence on the subject in question, this study has focused on the selection of quantitative studies. In the present study we have chosen not to establish temporal limits in the selection of studies. Also, as a consequence of the international collaboration carried out, we have expanded the number of languages for the search.

Before discussing the current findings, a caveat concerning this review should be noted. It is worth mentioning that, for some of the analyses, the subgroups included only two studies. The minimum number of studies to include in a meta-analysis has been discussed in the literature, without clear agreement [62]. As previous authors of meta-analysis claim, although the number of studies has a direct impact on statistical power and precision, if those few studies are relevant and their quality is high, it is worth including them. Similarly, other authors [63] argue that meta-analysis is always the best option to synthesize information as other alternatives “are likely to be based on less defensible assumptions and on less transparent processes” (p. 239). Nonetheless, we should bear in mind that analyses of subsamples will often be less precise because of the smaller numbers of studies [64].

One of the issues pointed out very frequently in studies on sexual abuse in people with disabilities is related to the difficulty of knowing the size of the problem, that is, data on its occurrence, prevalence and incidence. As mentioned earlier, previous studies reported prevalence rates between 7% and 34% for adults with intellectual disability [7]. This wide range reflects conceptual and methodological issues when it comes to knowing the occurrence of this phenomenon and its specific and differential impact on people with intellectual disability.

When paying attention to the publication date of the studies, it is possible to appreciate that prevalence decreases as the studies progress and the standard error also decreases, which could indicate that progressively more precise data are obtained.

In the present study, after assessing the methodological quality of the identified studies, and through the use of independent evaluators to code the information of interest, we have obtained more precise prevalence data. The overall prevalence was ·32.9%; the prevalence in women was 31.3% and the prevalence in men was 39.9%. Here, it is important to point out several aspects. First, data on sexual abuse in men with intellectual disability are scarcer than those on women and also present greater variability. For women, we were able to use 22 studies while for men, we only had 16. Second, differences could be related to gender roles. Here, given that men with intellectual disability tend to experience less overprotection than their female counterparts, the greater opportunities for involvement in the community without supervision could put them at higher risk of abuse. The finding that studies in non-clinical contexts have a higher prevalence of abuse in men than in women (37.7 vs. 28.4 vs.), would seem to support this explanation. Alternative explanations, also using the data obtained, could be related to the fact that men with intellectual disability are less supervised and, consequently, may be at higher risk in institutionalized or segregated contexts. In fact, overall prevalence of sexual abuse in institutions for women is 27.5% whereas for men is 50.8%.

According to our data, large institutions are the context in which sexual abuse is the most prevalent for men. This data agrees with previous findings which revealed that children and adolescents who grow up in institutional settings are affected by experiences of sexual abuse at a higher than average rate [65]. Likewise, peers are the most prevalent abusers. As studies almost unanimously indicate, abusers are mostly men. A recent study found that the prevalence of lifetime sexual violence, completed rape, and attempted rape against men with disabilities were comparable to those against women without disabilities [66]. In light of these findings, further studies are greatly advisable on the specific situation of residential care, as suggested by other authors [15].

Despite the slightly lower prevalence of sexual abuse in women with intellectual disability, the data remains very high. For example, if we compare these results with those obtained in national macro-surveys [67] where sexual violence and sexual abuse in females aged 16+ were 13.7% and 8.9%, respectively, with prevalence in the last 12 months of 1.3%, the disadvantaged situation of females with intellectual disability becomes clear.

This study confirms that higher severity of support needs is associated with higher prevalence, at least from mild to severe levels [23]. The comorbidity and additional medical support needs associated with profound levels of intellectual disability may explain its relatively lower prevalence. Alternative explanations could be related to the fact that those people with profound intellectual disability are not able to describe what has happened to them, as has been found in the literature [68]. In contrast to previous studies where age has been found associated to different prevalence [14,19], the current study has not found significant association between age and abuse. Possible explanation relates to the fact that the current meta-analysis only included studies on adult population. This was one of our objectives, in order to reduce the multifactorial nature of the abuse and the variables involved.

Some additional limitations should be noted. First, the studies included in the review come from convenience samples recruited from clinics or agencies serving individuals with disabilities, so data may not adequately represent the whole population with intellectual disability. More national and macro surveys are required. Further studies should also disaggregate data by disability, severity, gender, and other individual and contextual variables in order to be eligible for meta-analyses. Secondly, the studies reviewed are retrospective in nature and, although those in which the data suggest that sexual abuse occurred in adulthood have been selected, several studies report prolonged onset of abuse situations in earlier stages. These two factors could have introduced variability in the results obtained. Third, some studies could not be included in the quantitative synthesis, given the lack of specific and disaggregated data on the variables of interest. This could also influence the diversity of the evidence included. Fourth, it would have been very interesting to include sociodemographic characteristics as moderating variables, such as educational and social level, marital status, and employment status. Equally interesting would have been an inquiry into additional personal factors related to psychiatric comorbidity, or the presence and severity of behavioral problems. However, the absence of this information in the vast majority of published studies prevents the investigation of these features. Fifth, and in relation to this issue, the retrospective nature of the studies and the absence of prospective studies limits the possibility of drawing conclusions about risk factors for sexual abuse.

Before concluding, we also wish to underline that conducting a meta-analysis requires excluding studies of a qualitative nature and more phenomenological approaches. This does not mean rejecting the important contribution of these approaches. Nor does it imply denigrating the relevance of narrative reviews. However, the present meta-analysis fills a gap in the literature due to the selected methodological approach. This has required the rigorous selection of articles that met the inclusion criteria.

Finally, we recognize that it is a challenge to find a balance between providing individuals with intellectual disability with the necessary protective supervision so that they are not victims of abuse, while allowing them to enjoy their rights and freedom. The need to protect them from abuse cannot be based on the denial of a fundamental right: the right to live their sexuality. This topic should be approached in a multidimensional way with adequate supports to address their sexual development and sexuality [16]. This approach will empower them and will allow them to be included and participate in society while protecting them from the risk of abusive sexual behaviors.

## 5. Conclusions

Through a careful systematic review and meta-analysis of studies on sexual abuse in adults with intellectual disability, this study shows that one in three adults with this condition is a victim of sexual abuse as an adult. The abuse is more prevalent in males than in females, and it increases as the severity of the intellectual disability increase.

## Figures and Tables

**Table 1:** Summary details of studies included in the qualitative and quantitative synthesis.

First Author *	Year	Country	Context	Type of Study	Temporal Direction	Prevalence/ Incidence	Point/ Period	Length of Study
* Aker [28]	2020	Norway	clinical	Cross-sectional	Retrospective	PR	PE	2
* Allington [29]	2009	UK	clinical	Cross-sectional	Retrospective	PR	PO	
Beadle-Brown [30]	2010	UK	clinical	Longitudinal	Retrospective	PR	PE	0.8
Beail [31]	1995	UK	clinical	Cross-sectional	Retrospective	PR	PE	4
Brown [32]	1994	UK	clinical	Longitudinal	Retrospective	IN	PE	3
Buchanan [33]	1991	USA	clinical	Cross-sectional	Retrospective	PR	PO	
Cambridge [34]	2011	UK	clinical	Longitudinal	Retrospective	PR	PE	5
* Dickman [35]	2005	South Africa	clinical	Cross-sectional	Retrospective	PR	PE	10
* Dunne [36]	1990	Ireland	clinical	Cross-sectional	Retrospective	PR	PE	3
Elvik [37]	1990	USA	non-clinical	Cross-sectional	Retrospective	PR	PO	
Furey [38]	1994	USA	clinical	Cross-sectional	Retrospective	PR	PE	5
* Furey [39]	1994	USA	clinical	Case-control	Retrospective	PR	PE	5
* Furey [40]	1994	USA	clinical	Cross-sectional	Retrospective	PR	PE	5
Gil-Llario [41]	2018	Spain	non-clinical	Cross-sectional	Retrospective	PR	PO	
Gil-Llario [42]	2019	Spain	non-clinical	Cross-sectional	Retrospective	PR	PO	
Haydon [43]	2011	USA	non-clinical	Cross-sectional	Retrospective	PR	PE	1
Lewin [44]	2007	Sweden	clinical	Cross-sectional	Retrospective	PR	PO	
Lin [45]	2009	Taiwan	clinical	Longitudinal	Retrospective	PR/IN	PE	6
Majeed-Ariss [46]	2020	UK	clinical	Cross-sectional	Retrospective	PR	PE	1
McCabe [47]	1994	UK	non-clinical	Case-control	Retrospective	PR	PO	
McCarthy [48]	1997	USA	non-clinical	Cross-sectional	Retrospective	PR	PE	5
McCartney [49]	1998	Ireland	clinical	Cross-sectional	Retrospective	PR	PE	2
McCormack [50]	2005	Taiwan	clinical	Cross-sectional	Retrospective	PR	PO	15
Pan [51]	2007	UK	non-clinical	Cross-sectional	Retrospective	PR	PO	
Sequeira [52]	2003	Sweden	clinical	Case-control	Retrospective	PR	PO	
Shabalala [53]	2011	South Africa	clinical	Case-control	Retrospective	PR	PO	
Sobsey [54]	1991	Canada	clinical	Cross-sectional	Retrospective	PR	PE	2
Stoffelen [55]	2013	The Netherlands	clinical	Cross-sectional	Retrospective	PR	PO	
Turk [56]	1993	UK	clinical	Cross-sectional	Retrospective	PR	PE	3
Vadysinghe [57]	2017	Sri Lanka	clinical	Cross-sectional	Retrospective	PR	PE	5

* Excluded from quantitative synthesis due to lack of specific and disaggregated data on prevalence of sexual abuse of adults with intellectual disability.

**Table 2:** Prevalence of sexual abuse by selected variables.

Subgroups	Studies	Pooled Prevalence (%)	SE	*p*	Hete I^2^(%)	Q	*p*
Several	10	39.1 (21.1–57.1)	0.092	<0.001	100	3710.49	<0.001
Home	2	13.1 (−10.6–36.9)	0.121	0.278	89	9.09	<0.003
Institution	7	28.1 (12.0–44.1)	0.082	<0.001	99	450.19	<0.001
Service	3	34.3 (2.3–66.3)	0.163	0.036	99	226.23	<0.001
Place	22	32.7 (22.1–43.3)	0.054	<0.001	100	4767.23	<0.001
Professionals	3	17.6 (−1.9–37.0)	0.099	0.076	99	216.93	<0.001
Relatives	2	36.2 (−30.5–100)	0.340	0.287	100	353.80	<0.001
Peers	6	42.7 (19.7–65.7)	0.117	<0.001	99	516.21	<0.001
Several	7	25.4 (10.0–40.8)	0.079	0.001	98	336.73	<0.001
nonspec	4	39.2 (13.2–65.2)	0.133	0.003	100	2356.46	<0.001
Abuser	22	32.7 (22.1–43.3)	0.054	<0.001	100	4767.23	<0.001
Mild	9	24.3 (10.2–38.4)	0.072	<0.001	98	381.07	<0.001
Moderate	8	34.0 (14.7–53.4)	0.099	<0.001	99	849.21	<0.001
Severe	2	67.0 (59.5–74.4)	0.038	<0.001	44	1.79	0.180
Profound	2	18.6 (−15.7–53.0)	0.175	0.288	95	18.38	<0.001
Severity	21	31.7 (20.7–42.7)	0.056	<0.001	99	2545.31	<0.001
User	7	38.0 (20.1–55.8)	0.091	<0.001	99	403.47	<0.001
Other	10	26.8 (9.0–44.6)	0.091	0.003	99	917.27	<0.001
Both	5	37.4 (17.9–56.9)	0.100	<0.001	100	2596.75	<0.001
Informant	22	32.7 (22.1–43.3)	0.054	<0.001	100	4767.23	<0.001

Het. I^2^ = Heterogeneity statistic I^2^; Q = heterogeneity, Cochran’s Q.

## Data Availability

Raw data will be available upon request.

## Supplementary Materials

The following are available online at https://www.mdpi.com/1660-4601/18/4/1980/s1: Table S1: Summary of random-effects model for women, Table S2: Heterogeneity statistics of random-effects model for women, Figure S1: Forest plot of the prevalence of sexual abuse in women with intellectual disability, Table S3: Model results of the analysis for women, by clinical vs. non-clinical subgroup, Table S4: Heterogeneity statistics of random-effects model for women, by clinical vs. non-clinical subgroup, Figure S2: Forest plot of the prevalence of sexual abuse in women with intellectual disability, by clinical vs. non-clinical subgroup, Table S5: Model results of the analysis for women, by where the sexual abuse took place, Table S6: Heterogeneity statistics of the model for women by where the sexual abuse took place, Figure S3: Forest plot of the prevalence of sexual abuse in women with intellectual disability, by where the sexual abuse took place, Table S7: Model results of the analysis for women, by who were the abusers, Table S8: Heterogeneity Statistics of the model for women by who were the abusers, Figure S4: Forest plot of the prevalence of sexual abuse in women with intellectual disability, by who were the abusers, Table S9: Summary of random-effects model for men, Table S10: Heterogeneity statistics of random-effects model for men, Figure S5: Forest plot of the prevalence of sexual abuse in men with intellectual disability, Table S11: Model results of the analysis for men, by clinical vs. non-clinical subgroup, Table S12: Heterogeneity Statistics of the model for men by clinical vs. non-clinical subgroup, Figure S6: Forest plot of the prevalence of sexual abuse in men with intellectual disability, by clinical vs. non-clinical subgroup, Table S13: Model results of the analysis for men, by where the sexual abuse took place, Table S14: Heterogeneity Statistics of the model for men by where the sexual abuse took place, Figure S7: Forest plot of the prevalence of sexual abuse in men with intellectual disability, by where the sexual abuse took place, Table S15: Model results of the analysis for men, by who were the abusers, Table S16: Heterogeneity Statistics of the model for men by who were the abusers, Figure S8: Forest plot of the prevalence of sexual abuse in men with intellectual disability, by who were the abusers.

## Appendix A

**Table A1:** Quality assessment of the selected studies performed tools from the National Health, Lung, and Blood Institute (NHLBI) of the National Institutes of Health (NIH).

First Author	Year	C1	Study ^(1)^	C2	C3	C4	C5	C6	C7	C8	C9	C10	C11	C12	C13	C14	Quality Rating
Aker [28]	2020	Yes	Cross-Sectional	Yes	Yes	Yes	Yes	No	CD	CD	Yes	No	Yes	No	No	CD	Fair
Allington [29]	2009	Yes	Cross-Sectional	Yes	Yes	Yes	No	No	CD	Yes	Yes	No	No	CD	CD	CD	Fair
Beadle-Brown [30]	2010	Yes	Cross-Sectional	Yes	Yes	Yes	No	No	Yes	CD	Yes	No	Yes	No	No	CD	Fair
Beail [31]	1995	Yes	Cross-Sectional	Yes	Yes	Yes	No	No	Yes	CD	Yes	No	Yes	No	No	CD	Fair
Brown [32]	1994	Yes	Cross-Sectional	Yes	Yes	Yes	No	No	Yes	CD	Yes	No	Yes	No	No	CD	Fair
Buchanan [33]	1991	Yes	Cross-Sectional	Yes	No	Yes	Yes	Yes	CD	Yes	Yes	No	CD	CD	CD	CD	Fair
Cambridge [34]	2011	Yes	Cross-Sectional	Yes	Yes	Yes	No	No	Yes	CD	Yes	No	Yes	No	No	CD	Fair
Dickman [35]	2005	Yes	Cross-Sectional	Yes	Yes	Yes	No	No	Yes	CD	Yes	No	Yes	No	No	CD	Fair
Dunne [36]	1990	Yes	Cross-Sectional	Yes	Yes	Yes	No	No	Yes	CD	Yes	No	Yes	No	No	CD	Fair
ELvik [37]	1990	Yes	Cross-Sectional	Yes	No	Yes	Yes	Yes	CD	Yes	Yes	No	CD	CD	CD	CD	Fair
Furey [38]	1994	CD	Cross-Sectional	Yes	Yes	Yes	Yes	No	CD	Yes	CD	No	CD	No	No	CD	Fair
Furey [39]	1994	Yes	Case-control	Yes	Yes	Yes	Yes	Yes	CD	Yes	CD	Yes	CD	CD			Fair
Furey [40]	1994	Yes	Cross-Sectional	Yes	Yes	Yes	No	No	Yes	CD	Yes	No	Yes	No	CD	No	Fair
Gil-Llario [41]	2018	Yes	Cross-Sectional	Yes	Yes	Yes	No	No	Yes	Yes	Yes	No	Yes	No	No	CD	Fair
Gil-Llario [42]	2019	Yes	Cross-Sectional	Yes	Yes	Yes	No	No	CD	CD	Yes	No	Yes	No	No	CD	Fair
Haydon [43]	2011	Yes	Cross-Sectional	Yes	Yes	Yes	Yes	No	Yes	Yes	Yes	No	Yes	No	No	CD	Fair
Lewin [44]	2007	Yes	Cross-Sectional	Yes	No	Yes	Yes	Yes	CD	Yes	Yes	No	Yes	CD	CD	CD	Fair
Lin [45]	2009	Yes	Cross-Sectional	Yes	Yes	Yes	Yes	No	Yes	Yes	Yes	No	Yes	No	No	CD	Fair
Majeed-Ariss [46]	2020	Yes	Cross-Sectional	Yes	Yes	Yes	No	No	Yes	Yes	Yes	No	Yes	No	No	CD	Fair
McCabe [47]	1994	Yes	Case-control	Yes	No	Yes	Yes	Yes	CD	Yes	CD	CD	CD	CD			Fair
McCarthy [48]	1997	CD	Cross-Sectional	Yes	Yes	Yes	No	No	CD	Yes	Yes	No	Yes	No	No	CD	Fair
McCartney [49]	1998	Yes	Cross-Sectional	Yes	Yes	Yes	Yes	No	Yes	Yes	Yes	No	Yes	No	No	CD	Fair
McCormack [50]	2005	Yes	Case-control	Yes	Yes	Yes	No	No	Yes	Yes	Yes	No	Yes	No	No	CD	Fair
Pan [51]	2007	Yes	Case-control	Yes	Yes	Yes	No	No	CD	CD	Yes	No	Yes	No	No	CD	Fair
Sequeira [52]	2003	Yes	Case-control	Yes	Yes	Yes	Yes	Yes	CD	Yes	CD	Yes	CD	CD			Fair
Shabalala [53]	2011	Yes	Case-control	Yes	Yes	Yes	Yes	Yes	CD	Yes	CD	Yes	CD	CD			Fair
Sobsey [54]	1991	Yes	Cross-Sectional	Yes	Yes	Yes	No	No	CD	CD	Yes	No	Yes	No	No	CD	Fair
Stoffelen [55]	2013	Yes	Cross-Sectional	Yes	CD	Yes	No	Yes	Yes	No	Yes	No	Yes	No	No	No	Fair
Turk [56]	1993	Yes	Cross-Sectional	Yes	Yes	Yes	No	No	Yes	Yes	Yes	No	Yes	No	No	CD	Fair
Vadysinghe [57]	2017	Yes	Cross-Sectional	Yes	Yes	Yes	No	No	Yes	CD	Yes	No	Yes	No	No	CD	Fair

^(1)^ Cross sectional studies were evaluated with the Quality Assessment Tool for Observational Cohort and Cross-Sectional Studies, which includes 14 items; Case-control studies were evaluated with the Quality Assessment Tool for Case-Control Studies, which includes 12 items.

**Table A2:** Characteristics of included studies.

First Author	Year	Period of Publication	Context	Country	ID Abused	Total Sample	Women ID Abused	Tot Women	Men ID Abused	Tot Men	Age	Severity ID	Abuser	Informer
Beadle-Brown [30]	2010	3	non-clinical	UK	333	1926	NA	NA	NA	NA	38.9	moderate	Several	NA
Beail [31]	1995	2	non-clinical	UK	22	963	3	963	3	NA	29.5	mild	Home	other
Brown [32]	1994	1	non-clinical	UK	84	119	87	119	23	31	31	moderate	Several	user
Buchanan [33]	1991	1	non-clinical	USA	25	847	12	847	11	NA	28	moderate	Institution	other
Cambridge [34]	2011	3	non-clinical	UK	321	1857	116	6148	100	NA	34.02	moderate	Institution	other
Elvik [37]	1990	1	non-clinical	USA	13	35	5	35	NA	NA	24	profound	Institution	other
Furey [38]	1994	1	clinical	USA	171	461	123	461	46	46	30	moderate	Institution	user
Gil-Llario [41]	2018	3	non-clinical	Spain	22	360	2	360	5	180	NA	moderate	Service	other
Gil-Llario [42]	2019	3	non-clinical	Spain	125	360	35	360	53	180	50	moderate	Service	both
Haydon [43]	2011	3	non-clinical	USA	53	11878	33	53	20	5428	NA	mild	Several	both
Lewin [44]	2007	2	non-clinical	Sweden	23	42	6	7	1	10	50	moderate	Institution	other
Lin [45]	2009	3	non-clinical	Taiwan	1071	2005	0	2005	NA	NA	NA	NA	Several	both
Majeed-Ariss [46]	2020	3	non-clinical	UK	56	688	50	679	6	56	21.5	mild	Several	other
McCabe [47]	1997	2	non-clinical	Australia	8	30	0	30	NA	12	25.2	mild	Home	user
McCarthy [48]	1998	2	non-clinical	UK	70	185	43	185	30	120	NA	mild	Institution	user
McCartney [49]	2005	2	non-clinical	USA	10	494	6	494	6	296	NA	profound	Institution	other
McCormack [50]	2007	2	non-clinical	Ireland	74	118	39	118	NA	NA	NA	severe	Service	user
Pan [51]	2003	2	non-clinical	Taiwan	18	336	10	336	8	190	NA	mild	Several	user
Sequeira [52]	1991	1	non-clinical	UK	54	108	36	108	18	18	29.4	mild	Institution	both
Shabalala [53]	2013	3	non-clinical	South Africa	27	54	24	54	3	3	18	mild	Several	both
Sobsey [54]	1993	1	non-clinical	Canada	114	162	93	162	NA	30	19.2	severe	Several	other
Stoffelen [55]	2017	3	non-clinical	The Netherlands	10	21	0	21	10	19	40.5	mild	Several	user
Turk [56]	1993	1	non-clinical	UK	85	119	62	119	23	31	29	moderate	Several	other
Vadysinghe [57]	2017	3	clinical	Sri Lanka	NA	NA	74	82	8	82	28.3	mild	Home	NA

Period of publication: 1 = 1990–1994; 2 = 1995–2007; 3 = 2008–2020. NA = Not available.
