# Supplementary material for: Prevalence of Sexual Abuse in Adults with Intellectual Disability: Systematic Review and Meta-Analysis
Source: Int J Environ Res Public Health. 2021 Feb 18;18(4):1980. doi: 10.3390/ijerph18041980 (PMC7921934; doi:10.3390/ijerph18041980)
Supplement: Supplementary file 1 [file ijerph-18-01980-s001.pdf]

SUPPLEMENTAL MATERIAL

Table S1. Summary of random-effects model for women

Random-Effects Model (k = 22)

	Estimate	se	Z	p	CI Lower Bound	CI Upper Bound
Intercept	0.313	0.0640	4.89	< .001	0.187	0.438
	.	.	.	.	.	.

Note. Tau² Estimator: Empirical Bayes

Table S2. Heterogeneity statistics of random-effects model for women

Tau	Tau²	I²	H²	R²	df	Q	p
0.296	0.0879 (SE= 0.0278)	99.96%	2458.595	.	21.000	2200.978	< .001

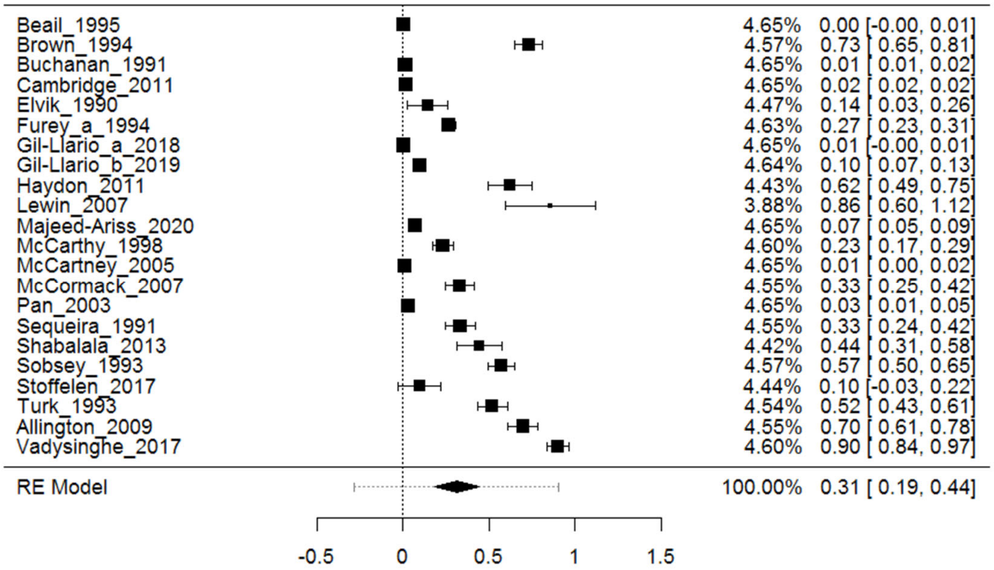

Figure S1. Forest plot of the prevalence of sexual abuse in women with intellectual disability

Table S3. Model results of the analysis for women, by clinical vs. non-clinical subgroup

Subgroups	Studies	Estimate	Lower bound	Upper bound	Std. error	p-Val
non-clinical	20	0.284	0.161	0.408	0.063	< 0.001
clinical	2	0.584	-0.039	1.207	0.318	0.066
Overall	22	0.313	0.187	0.438	0.064	< 0.001

Table S4. Heterogeneity statistics of random-effects model for women, by clinical vs. non-clinical subgroup

Studies	Q (df)	Het. p-Val	I^2
non-clinical	1321.084 (19)	< 0.001	99 %
clinical	269.701 (1)	< 0.001	100 %
Overall	2200.978 (21)	< 0.001	99 %

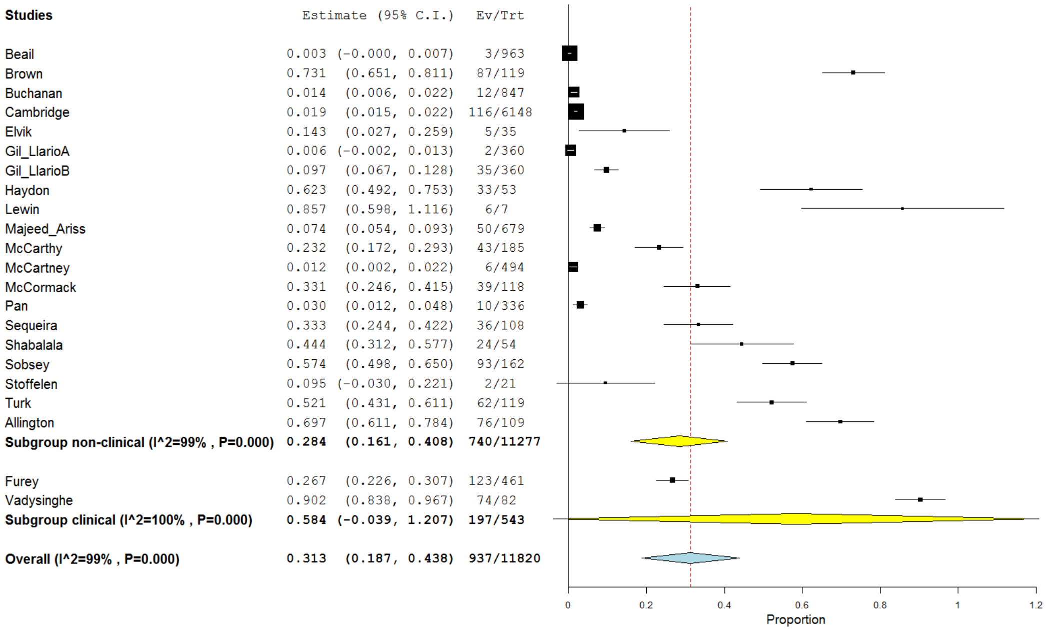

Figure S2. Forest plot of the prevalence of sexual abuse in women with intellectual disability, by clinical vs. non-clinical subgroup

Table S5. Model results of the analysis for women, by where the sexual abuse took place

Subgroups	Studies	Estimate	Lower bound	Upper bound	Std. error	p-Val
Home	2	0.452	-0.429	1.334	0.450	0.315
Several	8	0.385	0.191	0.578	0.099	< 0.001
Institution	9	0.275	0.081	0.468	0.099	0.005
Service	3	0.140	-0.048	0.328	0.096	0.143
Overall	22	0.313	0.187	0.438	0.064	< 0.001

Table S6. Heterogeneity statistics of the model for women by where the sexual abuse took place

Studies	Q (df)	Het. p-Val	I^2
Home	751.017 (1)	< 0.001	100 %
Several	626.388 (7)	< 0.001	99 %
Institution	524.049 (8)	< 0.001	98 %
Service	86.454 (2)	< 0.001	98 %
Overall	2200.978 (21)	< 0.001	99 %

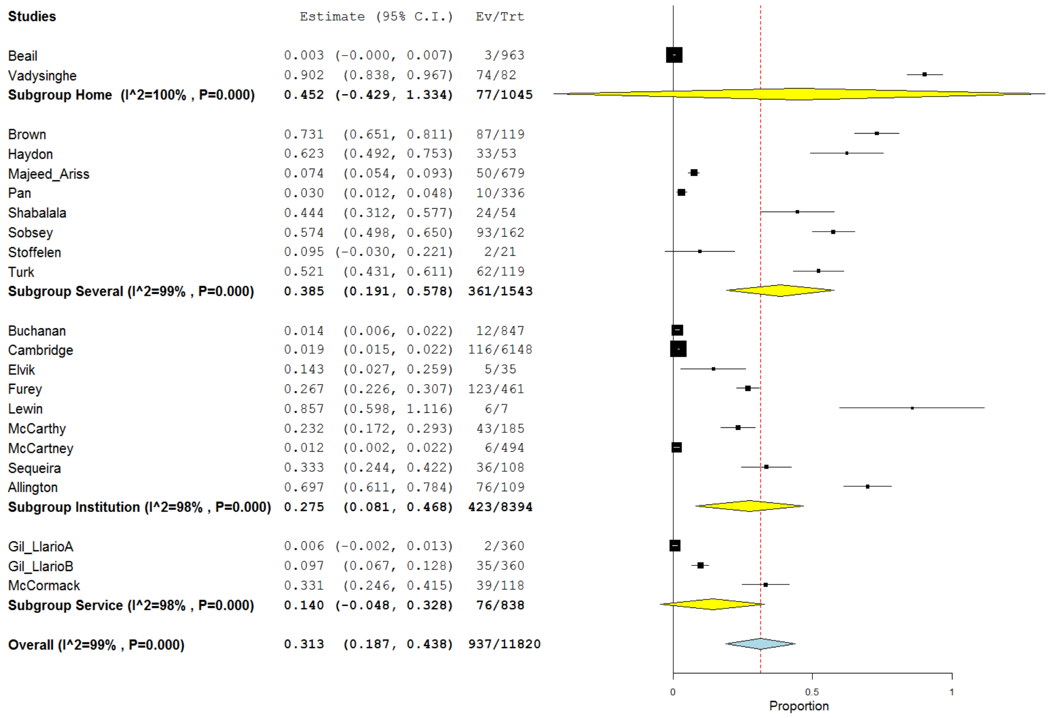

Figure S3. Forest plot of the prevalence of sexual abuse in women with intellectual disability, by where the sexual abuse took place

Table S7. Model results of the analysis for women, by who were the abusers

Subgroups	Studies	Estimate	Lower bound	Upper bound	Std. error	p-Val
Relatives	2	0.287	-0.272	0.847	0.285	0.314
Peers	8	0.441	0.218	0.665	0.114	< 0.001
Several	7	0.111	0.020	0.202	0.046	0.017
Professionals	2	0.064	-0.061	0.190	0.064	0.316
Non-specified	3	0.621	0.396	0.846	0.115	< 0.001
Overall	22	0.313	0.187	0.438	0.064	< 0.001

Table S8. Heterogeneity statistics of the model for women by who were the abusers

Studies	Q (df)	Het. p-Val	I^2
Relatives	215.524 (1)	< 0.001	100 %
Peers	1590.183 (7)	< 0.001	100 %
Several	135.550 (6)	< 0.001	96 %
Professionals	4.850 (1)	0.028	79 %
Non-specified	8.785 (2)	0.012	77 %
Overall	2200.978 (21)	< 0.001	99 %

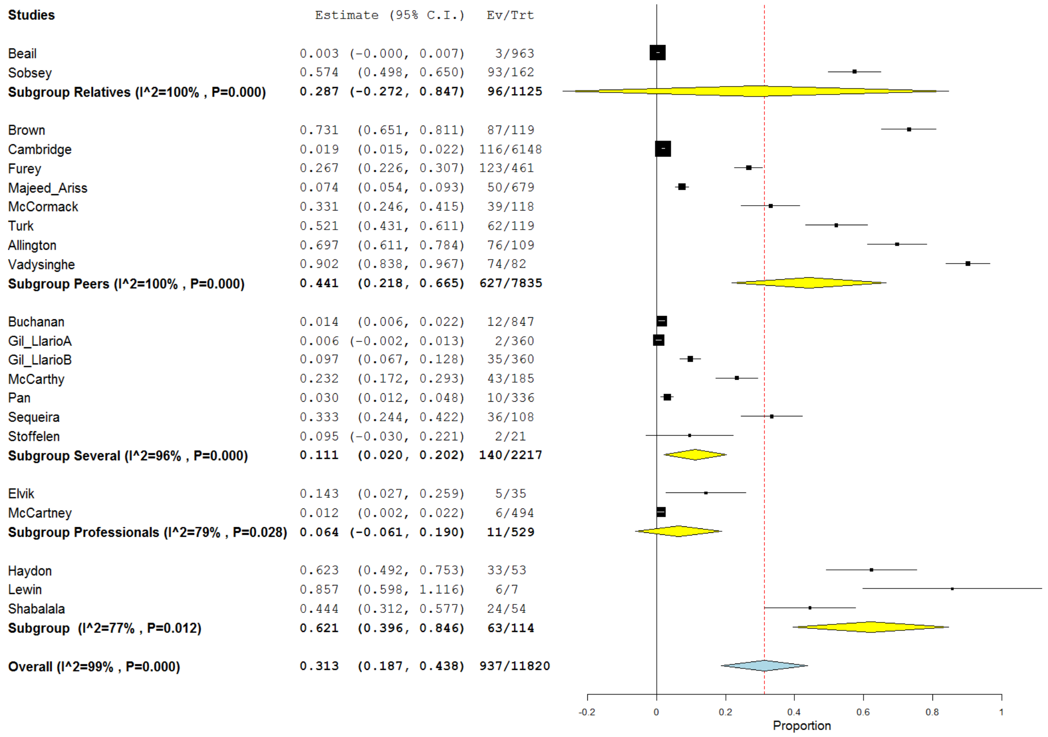

Figure S4. Forest plot of the prevalence of sexual abuse in women with intellectual disability, by who were the abusers

Table S9. Summary of random-effects model for men

Random-Effects Model (k = 16)

	Estimate	se	Z	p	CI Lower Bound	CI Upper Bound
Intercept	0.399	0.0937	4.26	< .001	0.215	0.583
	.	.	.	.	.	.

Note. Tau² Estimator: Empirical Bayes

Table S10. Heterogeneity statistics of random-effects model for men

Tau	Tau^2	I^2	H^2	R^2	df	Q	p
0.369	0.1364 (SE= 0.0513)	99.84%	638.725	.	15.000	5620.279	< .001

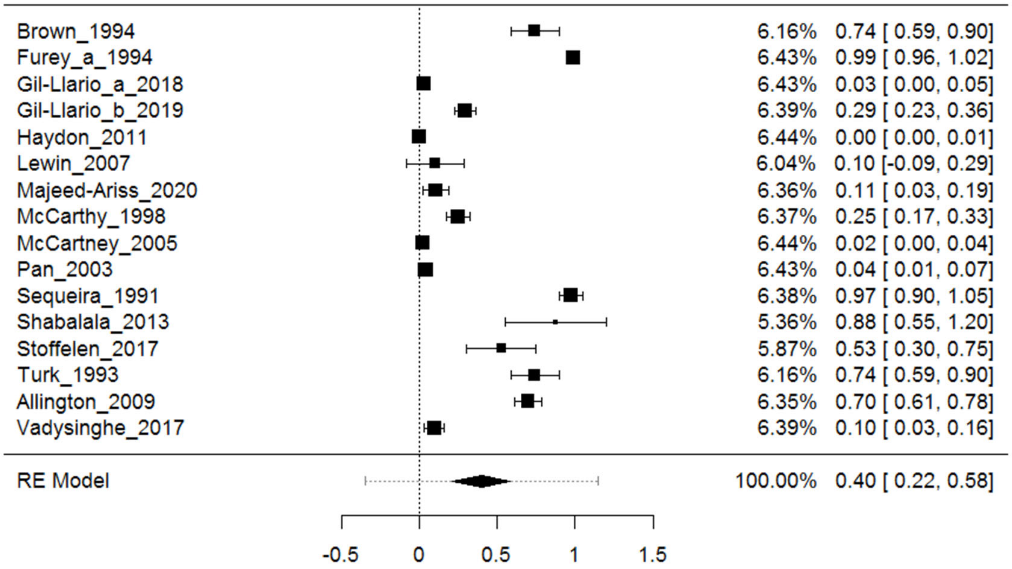

Figure S5. Forest plot of the prevalence of sexual abuse in men with intellectual disability

Table S11. Model results of the analysis for men, by clinical vs. non-clinical subgroup

Subgroups	Studies	Estimate	Lower bound	Upper bound	Std. error	p-Val
non-clinical	14	0.377	0.190	0.564	0.095	< 0.001
clinical	2	0.544	-0.330	1.418	0.446	0.223
Overall	16	0.399	0.215	0.583	0.094	< 0.001

Table S12. Heterogeneity statistics of the model for men by clinical vs. non-clinical subgroup

Studies	Q (df)	Het. p-Val	I^2
non-clinical	1301.434 (13)	< 0.001	99 %
clinical	612.892 (1)	< 0.001	100 %
Overall	5620.279 (15)	< 0.001	100 %

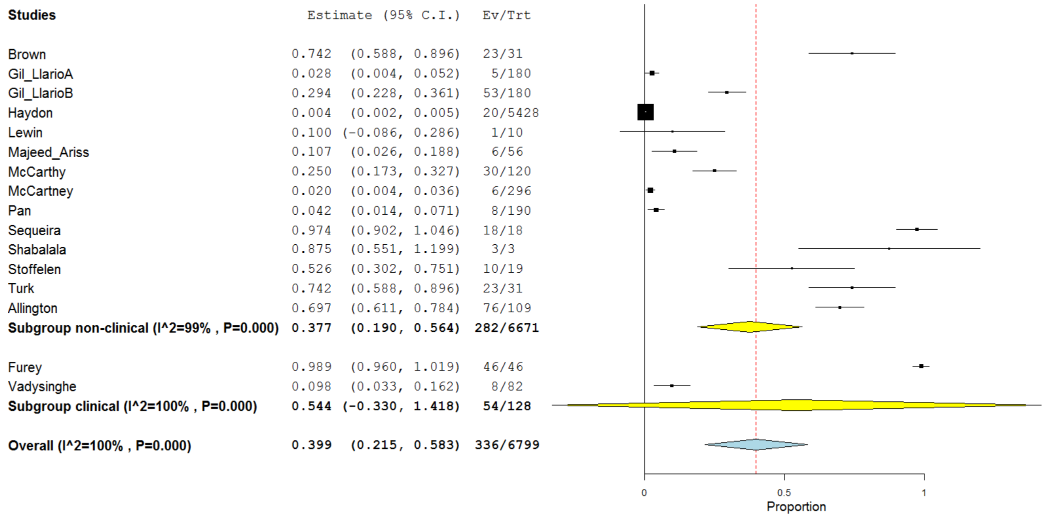

Figure S6. Forest plot of the prevalence of sexual abuse in men with intellectual disability, by clinical vs. non-clinical subgroup

Table S13. Model results of the analysis for men, by where the sexual abuse took place

Subgroups	Studies	Estimate	Lower bound	Upper bound	Std. error	p-Val
Home	1	0.098	0.033	0.162	0.033	NA
Several	7	0.418	0.141	0.694	0.141	0.003
Institution	6	0.508	0.158	0.858	0.179	0.004
Service	2	0.159	-0.102	0.421	0.133	0.232
Overall	16	0.399	0.215	0.583	0.094	< 0.001

Table S14. Heterogeneity statistics of the model for men by where the sexual abuse took place

Studies	Q (df)	Het. p-Val	I^2
Home	NA	NA	NA
Several	238.079 (6)	< 0.001	97 %
Institution	3703.365 (5)	< 0.001	100 %
Service	54.525 (1)	< 0.001	98 %
Overall	5620.279 (15)	< 0.001	100 %

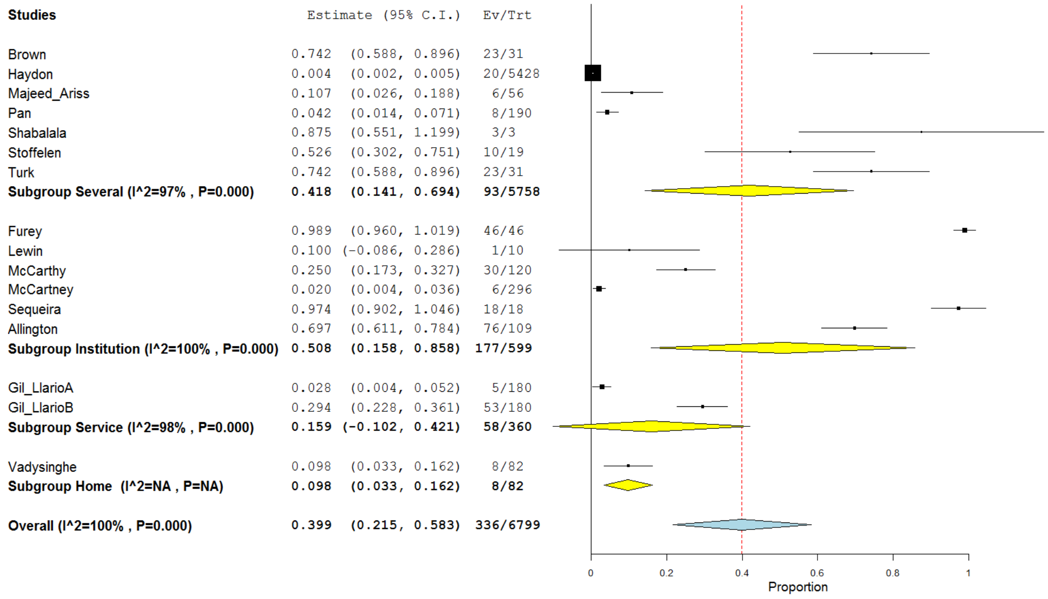

Figure S7. Forest plot of the prevalence of sexual abuse in men with intellectual disability, by where the sexual abuse took place

Table S15. Model results of the analysis for men, by who were the abusers

Subgroups	Studies	Estimate	Lower bound	Upper bound	Std. error	p-Val
Peers	6	0.561	0.263	0.859	0.152	< 0.001
Several	6	0.349	0.063	0.635	0.146	0.017
Professionals	1	0.020	0.004	0.036	0.008	NA
Non-specified	3	0.307	-0.224	0.839	0.271	0.257
Overall	16	0.399	0.215	0.583	0.094	< 0.001

Table S16. Heterogeneity statistics of the model for men by who were the abusers

Studies	Q (df)	Het. p-Val	I^2
Peers	895.241 (5)	< 0.001	99 %
Several	674.926 (5)	< 0.001	99 %
Professionals	NA	NA	NA
Non-specified	28.794 (2)	< 0.001	93 %
Overall	5620.279 (15)	< 0.001	100 %

Studies	Estimate (95% C.I.)	Ev/Trt
Brown	0.742 (0.588, 0.896)	23/31
Furey	0.989 (0.960, 1.019)	46/46
Majeed_Ariss	0.107 (0.026, 0.188)	6/56
Turk	0.742 (0.588, 0.896)	23/31
Allington	0.697 (0.611, 0.784)	76/109
Vadysinghe	0.098 (0.033, 0.162)	8/82
**Subgroup Peers (I^2=99% , P=0.000)**	**0.561 (0.263, 0.859)**	**182/355**
Gil_LlarioA	0.028 (0.004, 0.052)	5/180
Gil_LlarioB	0.294 (0.228, 0.361)	53/180
McCarthy	0.250 (0.173, 0.327)	30/120
Pan	0.042 (0.014, 0.071)	8/190
Sequeira	0.974 (0.902, 1.046)	18/18
Stoffelen	0.526 (0.302, 0.751)	10/19
**Subgroup Several (I^2=99% , P=0.000)**	**0.349 (0.063, 0.635)**	**124/707**
Haydon	0.004 (0.002, 0.005)	20/5428
Lewin	0.100 (-0.086, 0.286)	1/10
Shabalala	0.875 (0.551, 1.199)	3/3
**Subgroup (I^2=93% , P=0.000)**	**0.307 (-0.224, 0.839)**	**24/5441**
McCartney	0.020 (0.004, 0.036)	6/296
**Subgroup Professionals (I^2=NA , P=NA)**	**0.020 (0.004, 0.036)**	**6/296**
**Overall (I^2=100% , P=0.000)**	**0.399 (0.215, 0.583)**	**336/6799**

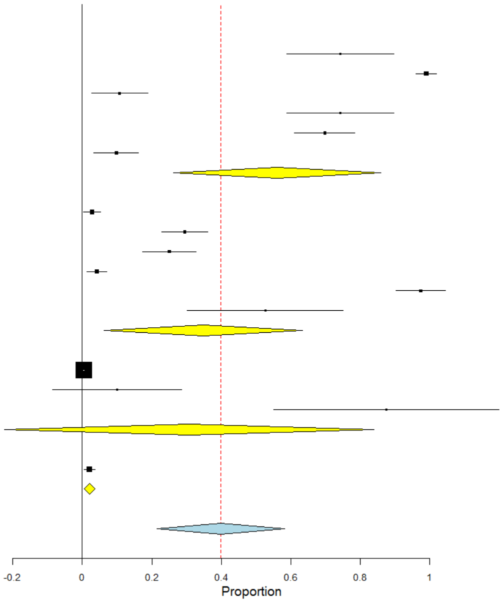

Figure S8. Forest plot of the prevalence of sexual abuse in men with intellectual disability, by who were the abusers
